# Emerging roles of circRNA in formation and progression of cancer

**DOI:** 10.7150/jca.30828

**Published:** 2019-08-28

**Authors:** Yuting Yin, Jiali Long, Qinglian He, Yuling Li, Yanqiu Liao, Peishan He, Wei Zhu

**Affiliations:** Department of Pathology, Guangdong Medical University, Dongguan 523808, Guangdong Province, China

**Keywords:** circular RNA, microRNA, cancer, colorectal cancer

## Abstract

Circular RNAs (circRNAs) are recently discovered as a special novel type of endogenous noncoding RNAs (ncRNAs), which form a covalently closed continuous loop and are highly represented in the eukaryotic transcriptome. Recent research revealed that circRNAs can function as microRNA (miRNA) sponges, regulators of splicing and transcription, as well as interact with RNA-binding proteins (RBPs). In this review, not only the function and mechanism, but also the experimental methods of circRNA are summarized. The summary of the current state of circRNA will help us in the discovery of novel biomarkers, the therapeutic targets and their potential significance in diagnosis and treatment of diseases. CircRNAs might play important roles in cancers especially in hepatocellular carcinoma, gastric carcinoma and colorectal cancer as well as serving as diagnostic or predictive biomarkers of some diseases and providing new treatments of diseases.

## Introduction

**Figure 1:**
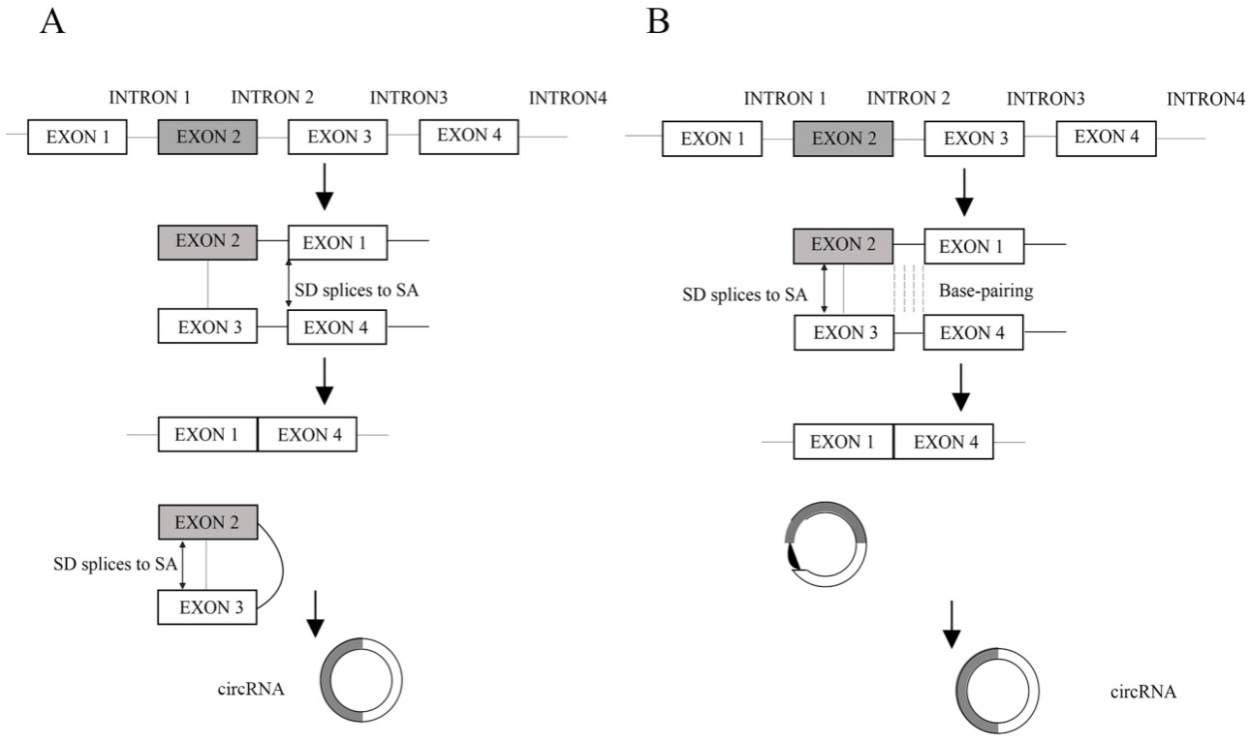
** A.** Lariat-driven circularization. Exon-skipping leads to a lariat whose restricted structure promotes circularization. **B.** Intron-pairing-driven circularization. Intron 1 and intron 3 are formed circular structure via base-pairing. Introns are removed or retained to form circRNA. (SD: splice donor, SA: splice acceptor).

Unlike linear RNAs that are terminated with 5′ caps and 3′ tails, circRNAs form covalently closed loop structures with neither 5′-3′ polarities nor polyadenylated tails, which makes them much more stable than linear RNA and insusceptible to degradation by RNA exonuclease or RNase R 1. CircRNA was first found in RNA viruses as early as the 1970s 2 and considered to be the result of the erroneously alternative splicing because of its low level expressions 3, 4. With the development of technology and bioinformatics, Jeck et al. put forward two models of circRNA formation 5. Model 1 is termed 'lariat-driven circularization' or 'exon skipping' (Figure 1A), and model 2 is termed 'intron-pairing-driven circularization' or 'direct back splicing' (Figure 1B) 5. Later, Zhang et al. discovered a new type of circRNA which is derived from introns and is termed circular intronic RNAs (ciRNAs) 6. Recently, Li et al. also revealed exons which are circularized with introns 'retained' between the exons and termed them EIciRNAs 7. In addition, the muscle blind protein (MBL) can bind to circMbl flanking introns to provoke the formation of circRNAs. In this way, MBL can act as RBPs (RNA-binding proteins, proteins that bind to RNA molecules, are found in the cytoplasm and nucleus, and they are important in forming ribonucleoproteins (RNPs), generally target single-stranded regions within secondary structure domains where the functional groups of the bases may be easily available for sequence specific recognition) to bridge two flanking introns close together 8. Furthermore, the interactions between RBPs form a bridge between the flanking introns, which bring the splice donor and splice acceptor into close proximity to promote circRNA biogenesis 9. According to the advances in our understanding of circRNA biogenesis, these properties of circRNAs can be summarized as follows. Firstly, circRNAs are much more stable than linear RNAs 1. Secondly, circRNAs are mainly composed of exons, which primarily reside in the cytoplasm and possibly have miRNA response elements (MREs) (miRNAs often bind their mRNA targets based on sequence complementarity in specific locations on the 3′ untranslated region (UTR) of the mRNA, termed MREs) 5, 10, 11. Thirdly, the abundance of circular molecules exceeds those of the corresponding linear mRNAs by >10-fold in some cases 5. Fourthly, circRNAs are evolutionarily conserved between different species 5, 10, 12. Lastly, the vast majority of circRNAs are endogenous noncoding RNAs (ncRNAs) 13.

## 1. The functins of circRNAs

### 1.1 CircRNAs function as miRNA sponges

The competitive endogenous RNAs (ceRNAs) can compete for miRNAs binding with their MREs 14. MiRNAs can prevent the translation of target mRNA by complementary pairing with target mRNA 3′-UTR region, which affects the stability of target mRNA and regulates the expression in the nucleus by binding to promoters 15, 16. Therefore, with its MREs, circRNAs, a new member of ceRNAs, plays an important role in the expression of RNAs by adsorbing miRNAs 17. It was tested that ciRS-7/cerebellar degeneration related protein 1 antisense (CDR1as) is shown to bind miR-7 18. Murine Sex-determining region Y (Sry) is the gene responsible for mammalian sex determination and can produce a circRNA which has 16 binding sites for miR-138, indicating that the circular Sry RNA likely acts as a miR-138 sponge 17, 19, 20. Additionally, Peng et al. found that cir-ZNF609 (ID: hsa_circ_0000615 in circBase) may act as a sponge for miR-150-5p to modulate the expression of AKT3 21.

### 1.2 CircRNAs regulate transcription and alternative splicing

Some research revealed that the knockdown of ciRNA derived from intron of ANKRD52 (Ci-ankrd52) leads to reduced expression of their parent genes by combinding RNA Pol II, and lines of evidence suggest one possible function for circRNAs as positive regulators of RNA Pol II transcription 6. Also, detailed studies discovered that circMbl is generated by the second exon of the splicing factor muscleblind, which competes with canonical premRNA splicing 8. Well, circMbl flanking introns and circMbl itself have conserved MBL binding sites, suggesting that general splicing factors, such as MBL, may have effects on alternative splicing that modulate the balance between circRNA biogenesis and canonical splicing 8.

### 1.3 CircRNAs interact with RBPs

Multiple evidence demonstrated noncoding RNA controls gene expression both at the transcriptional and post-transcriptional level through physical interaction with RBPs or other noncoding RNAs 22. It was demonstrated that ectopic expression of circ-Foxo3 (a circular RNA generated from a member of the forkhead family of transcription factors, Foxo3) repressed cell cycle progression by binding to the cell cycle proteins cyclin-dependent kinase 2 (also known as cell division protein kinase 2, CDK2) and cyclin-dependent kinase inhibitor 1 (CDKN1 or p21) 23. As a result, silencing endogenous circ-Foxo3 promotes cell proliferation 23. RNA-binding motif protein 20 (RBM20) is critical for the formation of a subset of circRNAs originating from the titin gene, which is known to undergo complex alternative splicing in mammalian hearts 24.

### 1.4 CircRNAs regulate translation

As a member of ncRNAs, few circRNAs can be translated. Evidences are presented based on electron microscopy and electrophoretic behaviour that hepatitis delta virus (HDV) contains a single stranded circular RNA molecule 25. This is the first animal virus identified with a circular RNA genome. Another interesting discovery is about covalently closed circular RNA (CCCRNA). It is the smallest one among all known viroids and virusoids and the only one that codes proteins 26. Its sequence possesses an internal ribosome entry site (IRES) and is directly translated through two (or three) completely overlapping ORFs (shifting to a new reading frame at the end of each round) 26.

## 2. Experimental methods of circRNAs

### 2.1 CircRNAs chip

The circRNAs chip (Arraystar Human circRNAs chip, ArrayStar) containing 5396 probes specific for human circular RNAs splicing sites can be used to investigate different expressions of circRNAs between tumor tissues and normal tissues. After hybridization and washing with samples, samples (tumor tissues and matched non-tumor tissues) were analyzed on the circRNAs chips. These chosen circRNAs are screened for further analysis^27^.

### 2.2 RNA extraction and microarray analysis

After being extracted from each sample using a homogenizer and TRIzol regent, total RNA is digested with RNase R to remove linear RNA and enrich circRNA. Subsequently, the enriched circRNA is amplified and transcribed into fluorescent cRNA (complementary RNA) utilizing a random priming method. After hybridizing, hybridized arrays are supposed to be scanned. When comparing two groups of profile differences, the fold change (i.e. the ratio of the group averages) between the groups for each circRNA is computed 28.

### 2.3 Quantitative real-time (qRT-PCR)

cDNA is synthesized with the Reverse Transcription System using random primers. qRT-PCR analysis is carried out to detect circRNAs expressions 29. After silencing and overexpression of circRNAs, the results of qRT-PCR are necessary to detect the expression of them.

### 2.4 Fluorescence in situ hybridization (FISH)

FISH can be used to investigate the expression and intracellular location of circRNAs in tissue and cell lines 29.

### 2.5 Cell Counting Kit-8 (CCK-8) and 5-ethynyl-2'-deoxyuridine (EDU)

Cell proliferation rates are detected with CCK-8 29 and EDU 30.

### 2.6 Gene ontology (GO) analysis

GO analysis (geneontology.org) can be used to construct meaningful annotation of gene products. GO contains three domains, including biological process (BP), cellular components (CC) and molecular function (MF) 28. The Gene Ontology, which provides the logical structure of the biological functions ('terms') and their relationships to one another, manifested as a directed acyclic graph the corpus of GO annotations, evidence-based statements relating a specific gene product (a protein, non-coding RNA, or macromolecular complex, which we often refer to as 'genes' for simplicity) to a specific ontology term.

### 2.7 Kyoto Encyclopedia of Genes and Genomes (KEGG) pathway analysis

KEGG is utilized to harvest pathway clusters covering the knowledge of the molecular interaction and reaction networks in genes producing differentially expressed circRNA 28. The higher order functional information is stored in the PATHWAY database, which contains graphical representations of cellular processes, such as metabolism, membrane transport, signal transduction and cell cycle. The KEGG databases are daily updated and made freely available (http://www. genome.ad.jp/kegg/).

### 2.8 The University of California Santa Cruz (UCSC) genome browser

Launched in 2001 to showcase the draft human genome assembly, the UCSC Genome Browser database (http://genome.ucsc.edu) and associated tools continue to grow, providing a comprehensive resource of genome assemblies and annotations to scientists and students worldwide 31. The UCSC Genome Browser database hosts a large repository of genomes with 166 assemblies from GenBank that represent over 93 different organisms across the tree of life 32.

### 2.9 CircNet Database

CircNet database (http://circnet.mbc.nctu.edu.tw/) provides the following resources: (i) novel circRNAs, (ii) integrated miRNA-target networks, (iii) expression profiles of circRNA isoforms, (iv) genomic annotations of circRNA isoforms (e.g. 282 948 exon positions), and (v) sequences of circRNA isoforms 33.

### 2.10 CircRNA Identifier (CIRI)

CIRI is able to unbiasedly and accurately detect circRNAs from transcriptome data by employing multiple filtration strategies. By applying CIRI to encode RNA-seq data, the prevalence of intronic/intergenic circRNAs as well as fragments specific to them in the human transcriptome can be identified and experimentally validated 34.

### 2.11 CircBank

The circular RNA database circBank was officially launched on July 7th, 2018. A total of 140,790 human circRNA records are recorded in the circBank database, which also develops a dedicated ID number, based on the name of its' Host gene and the corresponding location.

## 3. CircRNAs and cancers

### 3.1 CircRNAs and hepatocellular carcinoma (HCC)

Growing evidence indicates that circRNA expression alterations have a broad impact in biological characteristics of HCC. CircRNAs act as oncogenes or tumor suppressors in HCC. Furthermore, circRNAs interfere with hepatitis virus infection 35. Therefore, circRNAs can serve as potential diagnostic biomarkers for HCC 35. For instance, the oncogenic circRNA, CDR1as, is shown to be deregulated in a variety of cancers including HCC by acting as a sponge of miR-7 that sequesters and competitively inhibits the activity of miR-7 36. Liu et al. demonstrated that miR-7 can inhibit the growth of cancer cells and promote apoptosis^37^. It is known to all that target genes of miR-7 mainly including epidermal growth factor receptor (EGFR), AKT and so on, which are oncogenes or tumor suppressor genes in cancers^38^, ^39^. As for EGFR, it is highly expressed in pancreatic cancer, oral cancer, cervical cancer and so on^38^-^40^. The inhibition the expression of EGFR can strengthen the curative effect of chemoradiotherapy based on cisplatin^41^. In a word, ciRS-7 may act as a ceRNA of miR-7, competitively inhibiting the activity of miR-7 and promotes the expression of oncogenes. As a result, it can promote the initiation and development of cancer. We consider ciRS-7 as the target of the early diagnosis and therapy in cancer because the inhibition expression of ciRS-7 may affect the activities of multiple oncogenes. The expression of hsa_circ_0005986 is lower in HCC tissues compared with adjacent normal tissues as a tumor suppressor in HCC carcinogenesis 42. Especially, a total of 99 dysregulated circRNAs are identified to be associated with chronic hepatitis B (CHB) by circRNA/miRNA regulatory axes 43, 44. Among them, miR-122 is one of the most abundant miRNAs in the liver and plays a central role in the HCV life cycle 44. Recent studies indicate that circRNAs play a crucial role in controlling antiviral immune responses 45. In addition, Hsa_circ_0001649 is significantly downregulated in HCC, indicating that it may serve as a novel potential biomarker for HCC and may function in tumorigenesis and metastasis of HCC 46. Hsa_circ_0005075 can also act as a potential HCC biomarker because the expression of hsa_circ_0005075 correlates with HCC tumor size 47 (Table 1).

### 3.2 CircRNAs and gastric carcinoma (GC)

Present study finds that different expressions of circRNAs and the corresponding miRNAs interact through circRNA binding sites to regulate the expression of target genes 48. Results showed that a decrease in the circPVRL3 (Has_circ_0066779 is in gene symbol PVRL3 and it is named as circPVRL3) expression level is associated with the presence of GC and also with higher TNM stage and lower overall survival rates compared with that in adjacent noncancerous tissues 49. The receiver operating characteristic (ROC) curve can be used to investigate the diagnostic value of circPVRL3 in distinguishing GC tissues from adjacent nontumorous tissues and different TNM stages. It deserves to be mentioned that Kaplan-Meier overall survival curve shows that the survival time of patients with low expression is shortened. We are convinced that circPVRL3 may play a protection role in GC and can be applied as a powerful independent prognostic factor even a treatment target. In this study, a total of 9 miRNAs (miR-203, miR-1272, miR-1283, miR-31, miR-638, miR-496, miR-485-3p, miR-766, and miR-876-3p) and corresponding target mRNAs are predicted to have an interaction with circPVRL3. In addition, Zheng et al have reported that miR-203 can suppress invasion of GC cells by targeting ERK1/2/Slug/E-cadherin signaling^50^. Evidence showed that miR-31 can function as a suppressor regulated by epigenetic mechanisms and target integrin α5 suppressing tumor cell invasion and metastasis by indirectly regulating phosphatidylinositol 3-kinase (PI3K)/AKT pathway in human GC SGC7901 cells^51^, ^52^. Chen et al. characterized circPVT1 may promote cell proliferation by acting as a sponge for members of the miR-125 family. The level of circPVT1 is observed as an independent prognostic marker for overall survival and disease-free survival of patients with GC as well as a novel proliferative factor and prognostic marker in GC 53 (Table 2).

### 3.3 CircRNAs and colorectal cancer (CRC)

Bachmayr-Heyda A et al. were the first to report a global reduction of circular RNA abundance in CRC cell lines and tumor tissues compared to normal tissues, and they discovered a negative correlation of global circular RNA abundance and proliferation 54. Hsa_circ_0000069 knockdown can notably inhibit cell proliferation, migration, invasion, and induce G0/G1 phase arrest of cell cycle in vitro. It is demonstrated that hsa_circ_0000069, an important regulator in cancer progression, can be a promising target in the diagnosis and therapy in CRC 55. The expression of hsa_circ_001988 is significantly correlated with differentiation and perineural invasion, and hsa_circ_001988 may become a novel potential biomarker in the diagnosis of CRC and a potential novel target for the treatment of CRC 56. Zhu et al. conducted circular RNA profiles to identify circ-BANP as being enhance the growth of CRC cell by PI3K/Akt pathway. The biological function of circ-BANP (validated one circRNA generated from Exon 5-11 of BANP gene, termed circ-BANP) is convinced to be related with cell proliferation 29. What's more, the expression of hsa_circ_0007534 is significantly up-regulated in CRC tumor tissues compared with adjacent non-tumor tissues, and hsa_circ_0007534 expression is correlated with tumor stage and lymph node metastasis 57. Furthermore, the silence of hsa_circ_0007534 by siRNA significantly inhibited proliferation and induced apoptosis of CRC cells 57. Hsa_circ_0126897_CBC1 is thought to be a potential biomarker for CRC, and the cell cycle is closely associated with the occurrence and development of CRC using CapitalBio microarray technology 58. According to the study, 431 circRNAs are found differentially expressed in CRC tissues from patients with pulmonary metastasis compared with tissues without metastasis 28. Another study revealed that hsa_circRNA_105055 (upregulated), hsa_circRNA_086376 (downregulated) or hsa_circRNA_102761 (downregulated) may regulate the pulmonary metastasis of CRC through binding with miR-7 to regulate protein kinase C beta (PRKCB) that is involved in the NF-κB or Wnt signaling pathway 28 (Table 3).

## 4. Discussion and Conclusion

During the last decade, studies have convincingly documented that ncRNAs participate in regulating of cellular structure, function, and physiological development, and they may contribute to the pathogenesis and development of cancer. Among them, circRNAs are considered a new star in the field of ncRNAs research and extensively investigated. Considering the stability and cytoplasmic localization circRNAs, engineered circRNAs could be exploited for a range of molecular tools or therapies 9. Circular RNA constructs have been engineered both in vitro and in Vivo which could be applied to effectively sequester not only microRNAs or other RNAs of choice, but any RNA-binding protein with known sequence or structure specificity 9. In this review, we briefly summarize the characteristics and functions of circRNAs with emphasis on their functional role in biological processes associated with cancer. Firstly, circRNAs function as miRNA sponges. Secondly, circRNAs regulate transcription and alternative splicing. Thirdly, circRNAs interact with RBPs. Fourthly, circRNAs regulate translation. Taken together, these functions indicate that circRNAs have the potential to play important roles in transcription and post-transcription and to become ideal biomarkers in the diagnosis of diseases especially in cancer.

We also discuss the experimental methods of circRNAs for the further research. A better understanding of circRNAs in diseases may contribute to the development of novel detection methods, resulting more reliable diagnosis and treatment in cancer.

## Figures and Tables

**Table 1:** Overview of deregulated circRNAs in HCC

*CircRNA*	*Expression Change*	*Relative miRNA*	*Signal Path*	*Reference*
				
ciRS-7	up	miR-7	——	36
Has_circ_0005986	down	——	——	42
Hsa_circ_0001649	down	——	——	46
Hsa_circ_0079299	down	——	PI3K/AKT/mTOR	59
Circ_0067934	up	miR-1324	Wnt/β-catenin	60
Has_circ_0000567	down	miR-421	——	61
Circ_101368	up	miR-200a	HMGB1/RAGE	62
cSMARCA5	down	miR-17-3p/miR-181b-5p	——	63
Circ_0016788	up	miR-486	——	64
CircADAMTS13	down	miR-484	——	65
Circ-ZEB1.33	up	miR-200a-3p	——	66
Circ_100338	up	miR-141-3p	——	67
Circ_0008450	up	miR-548p	——	68
Has_circ_101280	up	miR-375	——	69
Has_circ_0103809	up	miR-490-5p	——	70
CircADAMTS14	up	miR-572	——	71

**Table 2:** Overview of deregulated circRNAs in GC

*CircRNA*	*Expression Change*	*Relative miRNA*	*Signal Path*	*Reference*
				
CircFAT1(e2)	down	miR-548g	——	72
CircPDSS1	up	miR-186-5p	——	73
Circ_100269	down	miR-630	——	74
Circ-DONSON	up	——	——	75
CircNRIP1	up	miR-149-5p	AKT1/mTOR	76
Has_circ_0001368	down	miR-6506-5p	——	77
Circ_LARP4	down	miR-424	——	78

**Table 3:** Overview of deregulated circRNAs in CRC

*CircRNA*	*Expression Change*	*Relative miRNA*	*Signal Path*	*Reference*
				
Circ-BANP	down	——	PI3 K/Akt	29
CircHIPK3	up	miR-7	c-Myb	30
Hsa_circ_0000069	up	——	——	55
Hsa_circ_001988	down	——	——	56
Hsa_circ_007534	up	——	——	57
CircITGA7	down	miR-370-3p	Ras	79
Circ_0026344	down	miR-21,miR-31	——	80
